# Eosinophils and Neutrophils Eliminate Migrating *Strongyloides ratti* Larvae at the Site of Infection in the Context of Extracellular DNA Trap Formation

**DOI:** 10.3389/fimmu.2021.715766

**Published:** 2021-08-12

**Authors:** Alexandra Ehrens, Nikolas Rüdiger, Lennart Heepmann, Lara Linnemann, Wiebke Hartmann, Marc P. Hübner, Minka Breloer

**Affiliations:** ^1^ Institute for Medical Microbiology, Immunology and Parasitology, University Hospital Bonn, Bonn, Germany; ^2^ Section of Molecular Biology and Immunology, Bernhard Nocht Institute for Tropical Medicine, Hamburg, Germany; ^3^ German Center for Infection Research (DZIF), Partner Site Bonn-Cologne, Bonn, Germany; ^4^ Department of Biology, University of Hamburg, Hamburg, Germany

**Keywords:** nematodes, L3, eosinophils, neutrophils, extracellular DNA traps, ETosis, *Strongyloides*

## Abstract

Parasitic nematodes such as hookworms actively penetrate the skin of their hosts, encountering skin-resident innate immune cells that represent the host´s first line of defense. Here we use *Strongyloides ratti* as a model for an intestinal helminth parasite with tissue migrating stages. We show that interception and killing of migrating larvae in mice during a 1^st^ infection occurred predominantly in skin and muscle tissue before larvae migrated *via* lung and head tissue to the intestine. Inhibition of larval migration was even more efficient in immune mice during a 2^nd^ infection where larvae barely left the site of entry i.e. the foot. Using cell-deficient mice we show that interception in the tissue was predominantly mediated by neutrophils and eosinophils while basophils and mast cells were dispensable *in vivo*. Likewise, neutrophils and eosinophils inhibited *S. ratti* L3 motility *in vitro* in the context of ETosis. Thereby eosinophils were strictly dependent on the presence of anti-*S. ratti* antibodies while neutrophils inhibited L3 motility as such. Also, MPO and MMP-9 were released by neutrophils in response to L3 alone, but immune plasma further stimulated MPO release in an antibody-dependent manner. In summary, our findings highlight the central role of the skin as first line of defense against helminth parasites in both, innate and adaptive immunity.

## Introduction

Strongyloids are parasitic nematodes that can, dependent on the species, infect humans, livestock and companion animals with a recently estimated global prevalence of 300 to 900 million people infected (1). Despite their importance for one health and economy, the protective immune response to this parasite is still not sufficiently understood. *Strongyloides ratti* is a natural parasite of wild rats that also patently infects mice and is used as a model for intestinal helminth parasites that display tissue migrating stages (2). Infectious third stage larvae (L3) may actively penetrate the skin of their rodent host and migrate within 2 days on yet not fully defined routes percutaneously, *via* muscle tissue, and partially also *via* the lung to the nasofrontal region of the head. After being swallowed, L3 arrive in the small intestine at day 3 post infection (p.i.). Here they mold *via* a L4 stage to parasitic female adults on day 5 to day 6 p.i., which live embedded in the mucosa (3–5). Immunocompetent mice clear the infection in the frame of a canonical type 2 immune response and remain semi-resistant to a 2^nd^ infection (2). To study the immunology and parasitology of *S. ratti* infections, a defined number of L3 is usually injected subcutaneously. Thereby, dependent on the mouse strain used, only 10 - 20% of *S. ratti* L3 successfully embed themselves in the small intestine during a 1^st^ infection and less than 5% during a 2^nd^ infection in immune mice (4, 6). This drastic loss in parasite numbers reflects the net effect of efficient interception of larvae during tissue migration and accelerated ejection of parasites from the intestine. However, the timing and the site of L3 killing in the tissue as well as the immune cells executing the killing and the mechanisms employed are still not fully understood.

The human pathogenic *Strongyloides stercoralis* cannot patently infect mice but may be implanted in diffusion chambers that allow cell ingress, to study the cellular and molecular requirements of L3 killing in the mouse system (7). These studies revealed a contribution of neutrophils and eosinophils and their respective effector molecules myeloperoxidase (MPO) and major basic protein (MBP) as well as larval opsonization *via* antibodies (Ab) and complement to the *in vivo* killing of L3 (7).

Neutrophils and eosinophils also share the feature of extracellular DNA trap cell death (ETosis) formation (8, 9). This cell death results in the explosive release of intracellular decondensed DNA that co-localizes with granules containing anti-microbial peptides (10, 11). As ETosis in response to migrating helminth parasites combines the trapping of migrating larvae with the direct delivery of effector molecules such as MPO and MBP to this “moving target”, it has become a recent focus of research in helminth immunology (10, 12–14). With regard to neutrophil ETosis (NETosis), *Onchocerca volvulus* nodules were shown to include DNA trap-releasing neutrophils. Here, NETosis is driven by the endosymbiotic *Wolbachia* bacteria released from the adult *O. volvulus* filariae and disappeared after *Wolbachia* depletion by doxycycline treatment (12). Furthermore, *Nippostrongylus brasiliensis* skin-penetrating L3 larvae induce NETosis. Interestingly, DNase II secretion by the *N. brasiliensis* L3 destroys the neutrophil DNA traps and thus, presents an immune evasion mechanism (14). Regarding *Strongyloides*, human and murine neutrophils were shown to immobilize *S. stercoralis* L3 *in vitro via* NETosis (13), while information on the rodent-specific *S. ratti* is missing so far. Release of extracellular DNA by eosinophils has been described in several eosinophil-associated diseases (9, 15) but only limited information is available on eosinophil ETosis (EETosis) in response to helminths. We showed recently that *Litomosoides sigmodontis* and *Dirofilaria immitis* microfilariae induce EETosis in human as well as murine eosinophils, which enabled larval entrapment and facilitated microfilariae clearance (10).

Here we use the simultaneous quantification of *S. ratti*-derived DNA and viable tissue-emigrating larvae to closely follow the migration path and fate of infectious larvae *in vivo.* We show that the majority of L3 are trapped and killed very efficiently in the skin and muscle tissue directly at the site of infection by neutrophils and eosinophils. Moreover, we demonstrate DNA trap formation by both, neutrophils and eosinophils, and show that both cell types inhibit larval motility *in vitro*. Thereby, opsonization of L3 by *S. ratti*-specific Ab derived from immune mice was required for eosinophil activity. Neutrophils, on the other hand, inhibited L3 larval motility as such in the context of MPO and matrix metalloproteinase-9 (MMP-9) release, with the release of the former one being enhanced by Ab. In conclusion, our findings highlight the central role of the skin as first line of defense against tissue-migrating infective larvae. Moreover, the results suggest different requirements for neutrophils and eosinophils to entrap L3 and indicate extracellular DNA trap formation as a part of granulocyte effector function during early larval tissue migration.

## Materials and Methods

### Ethics Statement

Animal experiments were conducted in agreement with the German animal protection law and experimental protocols were approved by Federal Health Authorities of the State of Hamburg (permission-numbers N111/16, A029/18 and A20/2020). All mice were bred in the animal facility of the BNITM and kept in individually ventilated cages under specific pathogen-free conditions. Mice were sacrificed by an overdosed CO_2_ narcosis followed by cervical dislocation in accordance with the German animal protection law. Mice for the bone marrow harvest were purchased from Charles River and sacrificed by an overdose Isoflurane followed by cervical dislocation.

### Mice

BALB/c mice, mast cell-deficient BALB/c Cpa3^Cre^ mice (16), basophil-deficient Mcpt8^Cre^ mice (17), and eosinophil-deficient ΔdblGATA [originally from The Jackson Laboratory (Bar harbor, ME, USA)] mice have been described before. For all experiments, male and female mice were used at 7 to 12 weeks of age, but experimental groups were matched for sex and age with maximally 7 days variance.

### Parasites and Infection

The *S. ratti* cycle was maintained in Wistar rats and L3 were purified from charcoal feces cultures of infected rats as described (18) (Available at http://www.wormbook.org). Infections were performed by s.c. infection of 2000 L3 in 30 µl PBS into the hind footpad of mice as described (6). Some mice received 350 µg anti Gr-1 (clone RB6-8C5, BioXcell, Lebanon, USA) one day before and at day 1 of *S. ratti* infection. Depletion of cells was verified by flow cytometry (**Supplementary Figure S1**).

### Quantification of *S. ratti* Parasites and *S. ratti*-Derived DNA in the Tissues

Whole feet, leg (shank and thigh)-derived skin and muscle tissue, skinned heads, lungs, small intestine and kidneys were prepared at indicated time points post *S. ratti* infection. For detection of viable parasites in small intestine, the intestine was opened longitudinally and washed with tap water briefly to remove feces. The intestine was covered with 20 ml tap water for 3 h at 37°C in a 50 mL Falcon Tube and shaken vigorously every hour. All other tissues were cut into small pieces and covered with tap water in 6-well plates (head, foot, skin and muscle) or 24-well plate (lung and kidney) and incubated for 3 h at 37°C with gently agitation every hour. Tissues were removed afterwards and emigrating parasites counted microscopically.

For DNA isolation tissue samples were cut into small pieces and incubated over night with 1 mL (foot), 2 mL (skin, muscle, kidney and lung) or 4 mL (small intestine and head) lysis buffer (0.1 M Tris, 5 mM EDTA, 2% SDS, 0.2 M NaCl) containing 100 μg/mL proteinase K (Roth, Germany) at 56°C. After centrifugation at 13000 x g for 10 minutes at 4°C, 0.5 mL (foot and lung) or 1 mL (skin, muscle, kidney, head and small intestine) supernatant was transferred into a fresh tube. Saturated NaCl to a final concentration of 25% was added, vortexed and incubated for 10 minutes at 4°C. After centrifugation at 13000 x g for 15 minutes at 4°C, 0.5 mL supernatant was harvested. 20 μg/mL RNase A was added and incubated for 15 minutes at 37°C. DNA was precipitated by addition of 500 μL isopropanol and centrifuged at 13000 x g for 5 minutes at 4°C. DNA was washed with 500 μL of 80% ethanol then allowed to dry at room temperature. The DNA was resuspended in 200 μL (foot, skin and muscle) or 400 μL (lung, head, kidney and small intestine) Millipore H_2_O. DNA concentration was adjusted to 5 ng/μL and used for quantification of *S. ratti-*derived DNA as described (6).

### Preparation of Cells From Skin Tissue for FACS Analysis

To analyze the skin-infiltrating cell populations, mice were infected as described above. After 3 and 6 h post infection the infected leg was shaved and amputated above the patella. As control, non-infected animals were taken. The skin was removed, minced and transferred to 10 mL DMEM buffer, containing 1 mg/mL Collagenase P (Sigma Aldrich, St. Louis, USA) and 1 U/mL DNAse I (Merck, Darmstadt, Germany). The tissue was shaken for 1 h at 37°C. After 30 min, the solution was pipetted up and down to increase digestion. Following incubation, the suspension was filtered through a 40 µm cell strainer and washed with DMEM containing 10% FCS. Cells were counted and used immediately for FACS staining. The cells were stained with Zombie Yellow (BioLegend, SanDiego, USA) for 20 min at RT, followed by surface antibody staining (CD45 AF700; 1:800 [clone DX5, BioLegend, SanDiego, USA], SiglecF PE 1:100 [clone 1RNM44N, Thermo Fisher Scientific GmbH, Germany], Ly6G APC-Cy7; 1:150 [clone 1A8, BioLegend, SanDiego, USA], Ly6C BV785; 1:150 [clone HK1.4, BioLegend, SanDiego, USA]) in Fc-Blocking buffer for 30 min at 4°C.

### Bone Marrow Isolation for Eosinophil and Neutrophil *In Vitro* Culture

For the neutrophil isolation and eosinophil-generation, BALB/c mice were sacrificed and the hind legs were isolated. The flesh was removed from the bones and the bones were opened at the far ends. The bone marrow was flushed out of the bones with RPMI, 10% FCS, L-glutamine and penicillin/streptomycin (Thermo Fisher Scientific GmbH, Germany) using a syringe and a 26 gauge needle. Collected bone marrow was filtered through a 70 µm filter (Miltenyi, Germany), cells were centrifuged at 400 x g, 10 min at 4°C and red blood cell lysis was performed from the cell pellet using a RBC lysis buffer (BioLegend, SanDiego, USA) for 5 min. The RBC lysis was stopped using RPMI medium and cells were counted using a CASY^®^ TT- cell counter (OMNI Life Science, Germany).

### Neutrophil Purification

Neutrophils were purified from bone marrow using Ly6G-MicroBeads and the MACS cell separation system according to manufacture procedure (Miltenyi, Germany). Therefore, bone marrow cells were incubated with Ly6G-Microbeads (Miltenyi, Germany) for 10 min in the fridge and added to a pre-equilibrated MS column in a magnetic field. The column was washed three times with MACS buffer (Miltenyi, Germany), the column was removed from the magnetic field and cells were flushed out of the column. The cell count was determined using the CASY^®^ TT- cell counter (OMNI Life Science, Germany) and the purity of the neutrophils was analyzed using flow cytometry. Therefore, cells were stained for Gr1-PeCy7 (BioLegend, SanDiego, USA).

### Eosinophil Generation

Bone marrow from naïve mice were used to generate bone marrow-derived eosinophils as previously described (10). Therefore, isolated bone marrow was counted using the CASY^®^ TT- cell counter system and cells were seeded in in Advanced RPMI medium with 20% FBS, 1% penicillin/streptomycin, 0.1% gentamycin, 2.5% HEPES and 1% Glutamax (Thermo Fisher Scientific GmbH, Germany). Cells were cultured with Stem cell factor (SCF) and FMS-like tyrosine kinase 3 ligand (FLT3L) (Peprotech, Rocky Hill, USA) for the first 4 days. Afterwards the growth factors were exchanged with IL-5 (Peprotech, Rocky Hill, USA). Half of the medium was exchanged every other day and on day 8, the cell culture flask was exchanged. After 12 days, cells were harvested and checked for the eosinophil purity using flow cytometry. A CytoFLEX Flow Cytometer (Beckman Coulter, Brea, USA) was used to analyze the purity of the eosinophils using anti-SiglecF-APC-Cy7 (BD Bioscience, San Jose, USA) antibodies.

### Antibody-Depletion From Plasma

Blood was harvested from either naïve mice or mice that received two *S. ratti* infections 4 weeks apart 14 days after the 2^nd^ infection by cardiac puncture. Cells were separated by centrifugation and plasma was depleted for antibodies using the ChroSpin, Protein A/G (Dalex Biotech GmbH) according to manufacturer’s protocol. Therefore, 400 µL of the sanitized 50% slurry was added to a column. 200 µL plasma was added to the equilibrated resin and incubated for 3 min with end-over-end mixing. The columns were centrifuged for 1 min at 1000 x g and flushed plasma was collected. For washing, 500 µL wash buffer was added, inverted and centrifuged twice. Column was placed in a clean tube and elution buffer was added, tubes were inverted and centrifuged. Elution was repeated and neutralization buffer was added to the elution fraction. Antibody-depletion was repeated with collected plasma from the first depletion round.

### L3 Motility Assessment

Larval motility was assessed in co-cultures with neutrophils and eosinophils. L3 were harvested from the feces charcoal cultures of infected rats as described (18) and washed three times in PBS supplemented with penicillin and streptomycin (100 U/mL) (Thermo Fisher Scientific GmbH, Germany). Larvae were resuspended in RPMI medium with 10% FCS, 1% penicillin/streptomycin, 0.1% gentamycin, 2.5% HEPES and 1% Glutamax (Thermo Fisher Scientific GmbH, Germany). L3 were picked under microscopic control and cultured in 96-well flat bottom plates at a concentration of 30 L3 per well at 37°C and 5% CO_2_ for 24 h to regain full motility. Subsequently 100,000 isolated neutrophils or generated eosinophils were added to the L3 culture. Therefore, MACS-purified neutrophils or bone marrow-derived eosinophils were resuspended in RPMI medium with 10% FBS, 1% penicillin/streptomycin, 0.1% gentamycin, 2.5% HEPES and 1% Glutamax. Cells were added to a 96-well flat bottom plate and co-cultured with 30 L3. In addition, in some wells the medium was supplemented with 1% plasma from naïve or *S. ratti*-infected mice. The motility was assessed every day for up to 3 days using a 5 x magnification, respectively. Flowing scoring system ranging from 0 to 4 was applied: L3 having a score of 4 showed fast and continuous movement, while a L3 having a score of 3 showed slower but continuous movements. A score of 2 indicated slow and discontinuous movements, while L3 with a score of 1 only showed sporadic movements at the ends. In case no movement was recorded for the L3, a score of 0 was assigned. The motility scores were performed in a blinded manner.

### Fluorescence Microscopy

For fluorescence microscopy, cover glass slides (170 +/- 5 µm) were added to a 24-well plate and coated for 1 h with 0.1% polyethylineimine in water and washed three times in dH_2_O for 1 h each. Afterwards slides were air dried and then sterilized under UV light for at least 20 min. 200,000 purified neutrophils or eosinophils in RPMI medium with 10% FBS, 1% penicillin/streptomycin, 0.1% gentamycin, 2.5% HEPES and 1% Glutamax were added to the slides. 100 L3 were added in the absence or presence of 1% plasma from naïve or infected animals. In addition, some cells were stimulated with 50 ng/mL PMA (Sigma Aldrich, St. Louis, USA). Cells were incubated for 24 h at 37°C and 5% CO_2_, PFA (Sigma Aldrich, St. Louis, USA) was added to a concentration of 2%, plates were centrifuged, supernatant was removed and fresh 4% PFA was added for 20 min at RT. Afterwards, glass slides were washed with PBS (Thermo Fisher Scientific GmbH, Germany) and then blocking buffer was added (10% normal goat serum (Thermo Fisher Scientific GmbH, Germany), 0.3% Triton-X in in 0.3 M NaCl, 0.03 M sodium citrate, pH 7 buffer) and incubated overnight at 4°C. Staining was performed the next day using 0.3 M NaCl, 0.03 M sodium citrate, pH 7, with the unconjugated primary antibody against citrullinated H3 histone (Abcam, Germany) (1/500 diluted) overnight at 4°C. Glass slides were washed trice with PBS for 5 min each, and then the conjugated antibodies were added in the 0.3 M NaCl, 0.03 M sodium citrate, pH 7 buffer. The secondary Alexa Fluor 488 (AF488) goat anti-rabbit IgG (H+L) antibody (Thermo Fisher Scientific GmbH, Germany) (1/500 diluted) was incubated for 1.5 h at RT in the dark. Slides were washed trice with PBS. Afterwards, 0.5 µM Sytox orange (Thermo Fisher Scientific GmbH, Germany) in 0.3 M NaCl, 0.03 M sodium citrate, pH 7 buffer was added for 20 min at RT. Slides were then washed in distilled H_2_O and mounted with moviol (Carl Roth, Germany) and anti-fade (Thermo Fisher Scientific GmbH, Germany) (diluted 1/50). Samples were analyzed using the Axio Observer 7 from Zeiss with the Zen2.6 software (Carl Zeiss, Germany).

### ELISA

For the ELISA, the supernatant of the co-cultures was collected after 24 h and stored at -20°C until further use. Supernatant from neutrophil cultures were analyzed for MPO and MMP-9. Therefore, MPO ELISA from R&D Systems (Catalog Number: DY3667) and MMP-9 ELISA from R&D Systems (Catalog Number: DY6718) were used according to manufacturer’s instructions. Briefly, capture antibody for MPO or MMP-9 was diluted in PBS (1/180) and added to 96-half area plates and incubated over night at 4°C. Afterwards, plates were washed three times using PBS with 5% Tween and plates were blocked with PBS/1% BSA for 2 h at RT. Blocking was removed and a serial dilution of MPO or MMP-9 standard, PBS/1% BSA as a negative control and diluted (1/2 for MMP-9 and 1/20 for MPO) supernatant was added to the plates for 2 h at RT. Plates were washed 5 times with PBS and 5% Tween for 1 min each and the detection antibody (1/180 diluted in PBS/1% BSA) was added and incubated for 2 h at RT. Plates were washed 5 times and Strep-HRP diluted in PBS/1% BSA was added and incubated for 1 h at RT. Detection for all ELISAs was done by the addition of TMB until color change was observed, the reaction was stopped using 1 M H_2_SO_4_ and plates were measured using the SpectraMax 190 plate reader (Molecular Devices LLC) at 450 nm.

### Statistical Analysis

All data were assessed for normality and groups were compared using Student`s t-test (parametric, 2 groups at one time point) or 2-way ANOVA (parametric, 2 groups over time) or Kruskal-Wallis (nonparametric, more than 2 groups) using GraphPad Prism software (San Diego) as indicated in the figure legends. P values of < 0.05 were considered to indicate statistical significance. Asterisks or symbols as indicated in the figure legends indicate statistically significant differences *p < 0.05; **p < 0.01; ***p < 0.001.

## Results

### Efficient Elimination of Migrating *S. ratti* Larvae at the Site of Infection

Information regarding the exact migration route and kinetics of parasitic intestinal nematodes is often limited to snap shots of defined organs and time points such as lung and intestine at days 1-2 and 5-6 post infection. Moreover, the exact quantification of tissue-migrating larvae can be compromised by the fact that especially the larvae that are efficiently trapped by tissue-resident immune cells cannot emigrate out of tissue samples incubated in the standard emigration assay (19).

**Figure 1:**
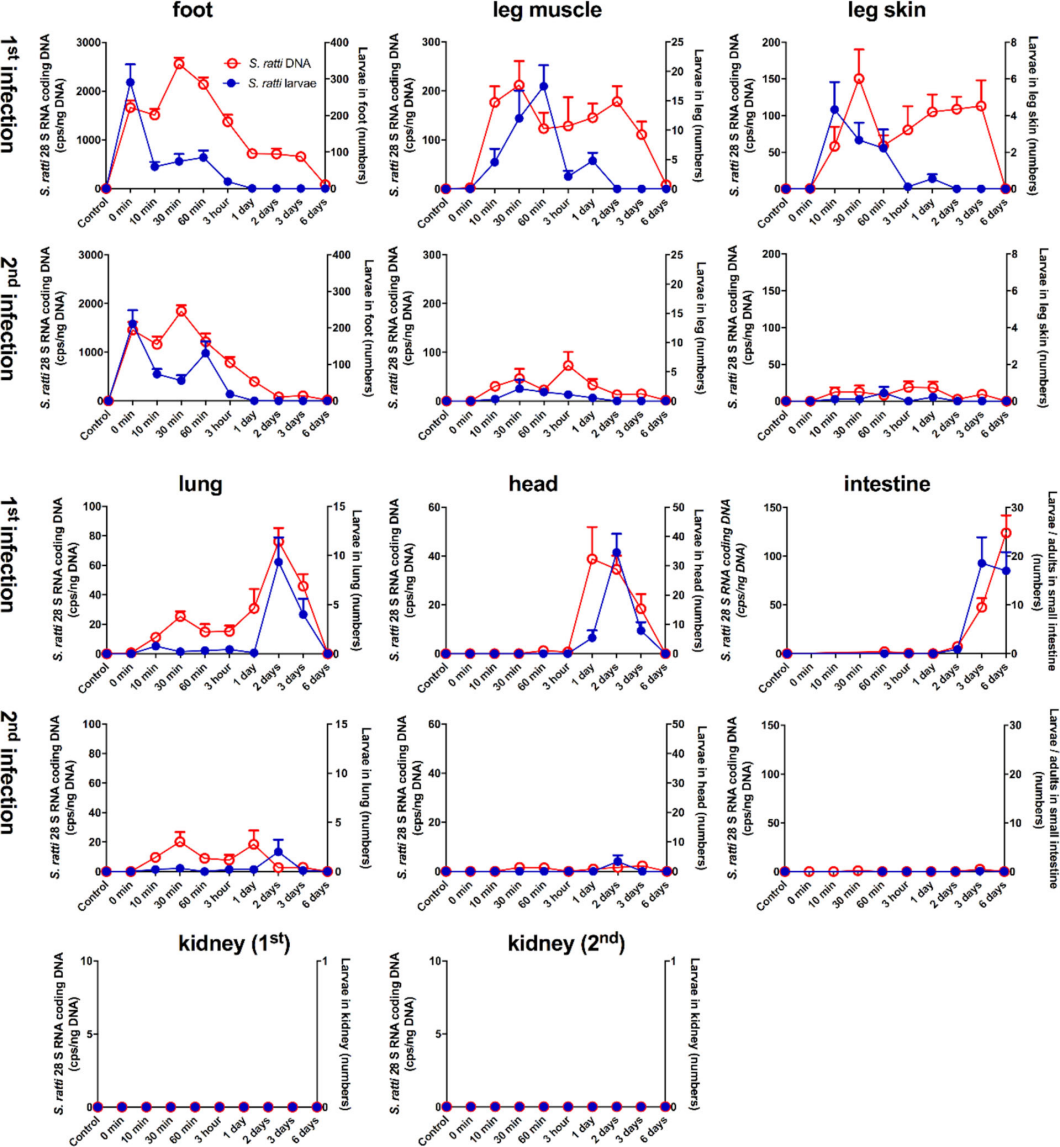
Kinetic analysis of *S. ratti* parasite burden in infected mice. Naïve BALB/c (1^st^ infection) mice and immune BALB/c (2^nd^ infection) mice were infected with 2000 *S. ratti* L3 by s.c. injection into the footpad and sacrificed at the indicated time points. *S. ratti*-derived DNA (open red circles - left ordinate) was quantified in the indicated tissues by qPCR. Viable *S. ratti* L3, L4 and adults (filled blue circles - right ordinate) that emigrated out of indicated tissues were counted. Graphs show mean of combined results derived out of 2 independent experiments with 3 - 5 mice per time point, group and individual experimental repeat, error bars show SEM. *S ratti* DNA in blood and spleen was investigated as well and shown in **Supplementary Figure S2**. Lung tissue damage after *S. ratti* migration is shown in **Supplementary Figure S3**.

We intended to closely follow the fate of migrating *S. ratti* L3 from the site of infection to the intestine in naïve mice encountering the 1^st^ infection and in immune mice encountering a 2^nd^ infection. To this end, mice were infected by s.c. injection of *S. ratti* L3 into the footpad and sacrificed at different time points to analyze parasite burden in different tissues “on their way” from the infection site to the intestine. We quantified L3 that are viable and still able to migrate, performing standard emigration assays (**Figure 1** blue circles). To additionally detect dead and/or trapped L3, we have developed a gDNA-based qPCR for the *S. ratti* 28S RNA gene (6) allowing us to quantify *S. ratti*-derived DNA within the tissue samples (**Figure 1** red circles).

During a first infection, *S. ratti*-derived DNA and viable L3 were present after the s.c. injection in the footpad. However, although mice were sacrificed immediately after infection (0 min) and no L3 were present in the surrounding tissues, only 200-300 L3 that is 10% of the initial infection dose could be retrieved. The numbers of viable L3 in the foot rapidly declined within 10 minutes and no viable, migrating L3 were present at day 1 p.i. or later. By contrast, *S. ratti*-derived DNA in the foot peaked at 30 min p.i. and was detected until day 3 p.i., and with very low copy numbers until day 6 p.i., suggesting that this remaining *S. ratti*-derived DNA originated from either immobilized and/or killed and degrading *S. ratti* L3. Analysis of leg-derived skin and muscle tissues revealed presence of *S. ratti*-derived DNA and viable, migrating L3 at 10 min after injection of L3 i.e. the time point when the migrating L3 had left the foot. Numbers of viable L3 declined in the leg after 60 min p.i., whereas *S. ratti*-derived DNA was detected until day 3 p.i.. This remaining *S. ratti-*derived DNA again most likely reflected the presence of either immobilized and/or dead *S. ratti* L3 that were present for several days after the rapid emigration of viable L3 from the leg. *S. ratti*-derived DNA was absent in spleen and whole blood of 3 and 6 h-infected mice (**Supplementary Figure S2**), suggesting that L3 did not use the peripheral blood circulation for migration to the lungs or head. Analysis of lung- and head-derived tissues showed viable L3 were first present 2 days (lung) or 1 day (head) after the infection with maximal numbers in both tissues at day 2 p.i. and rapid decline in numbers at day 3 p.i. Of note, *S. ratti*-derived DNA was maximal in lung and head at day 2 p.i. that is simultaneously with the viable L3 and also declined simultaneously with the numbers of viable L3, suggesting that no immobilized and/or dead L3 remained in lung and head tissue. Viable *S. ratti* L3 immigrated from day 3 p.i. onwards into the small intestine and the numbers of parasites, now molted to adults, peaked at day 6 p.i.. Also *S. ratti*-derived DNA was detectable from day 2 p.i. onwards in the intestine and peaked day 6 p.i.. Analysis of kidney-derived tissue revealed no detectable *S. ratti*-derived DNA or viable L3 throughout infection.

The analysis of parasite burden in the feet of immune mice encountering a 2^nd^ infection closely resembled mice that were infected for the first time. Again, the numbers of viable L3 were maximal immediately after the injection and rapidly declined within 10 minutes. *S. ratti*-derived DNA was maximal between 10 and 60 min p.i. and declined until day 2 p.i. with only very low copy numbers present until day 6 p.i.. By contrast, analysis of leg-, lung- and head-derived tissues revealed drastically reduced numbers of viable L3 and reduced copy numbers of *S. ratti-*derived DNA compared to a 1^st^ infection. Viable L3, L4, adults or *S. ratti*-derived DNA were not detectable in the small intestine of immune mice. Consistent with the results of the 1^st^ infection, no viable L3 or *S. ratti*-derived DNA was detectable in kidney-derived tissues throughout the infection.

In summary, these results show that *S. ratti* L3 did not migrate randomly through their host`s tissue and body fluids, but rather followed a specific route from the site of entry to the intestine. L3 were first present in the foot, 10 minutes later in the leg-derived tissues and 2 days later in lung and head to finally arrive at the small intestine at day 3 p.i.. Thereby, other tissues such as the kidney remained parasite-free. *S. ratti*-derived DNA was detected for a noticeable longer time period in foot and leg-derived tissues than viable L3, suggesting that the local trapping and/or killing of L3 predominantly occurred in these tissues and not in the lung and the head where migrating L3 and *S. ratti*-derived DNA declined simultaneously. In immune mice encountering a 2^nd^ infection, L3 were directly retained and eliminated at the site of re-infection, as almost no migrating *S. ratti* L3 nor *S. ratti*-derived DNA were detectable in other tissues than the foot. This finding highlights the importance of the skin and its surrounding tissue as first line of defense in immune mice, preventing other organs from infection-induced pathology. Accordingly, the drastic difference in lung parasite burden during 1^st^ and 2^nd^ infection was reflected by lung pathology. Mice infected for the first time showed macroscopic hemorrhagic lung lesions and elevated erythrocyte numbers in BAL compared either to uninfected control mice or to immune mice encountering a 2^nd^ infection (**Supplementary Figure S3**).

### Tissue-Migrating *S. ratti* L3 Are Eliminated by Eosinophils and Granulocytes While Mast Cells and Basophils Are Dispensable *In Vivo*

**Figure 2:**
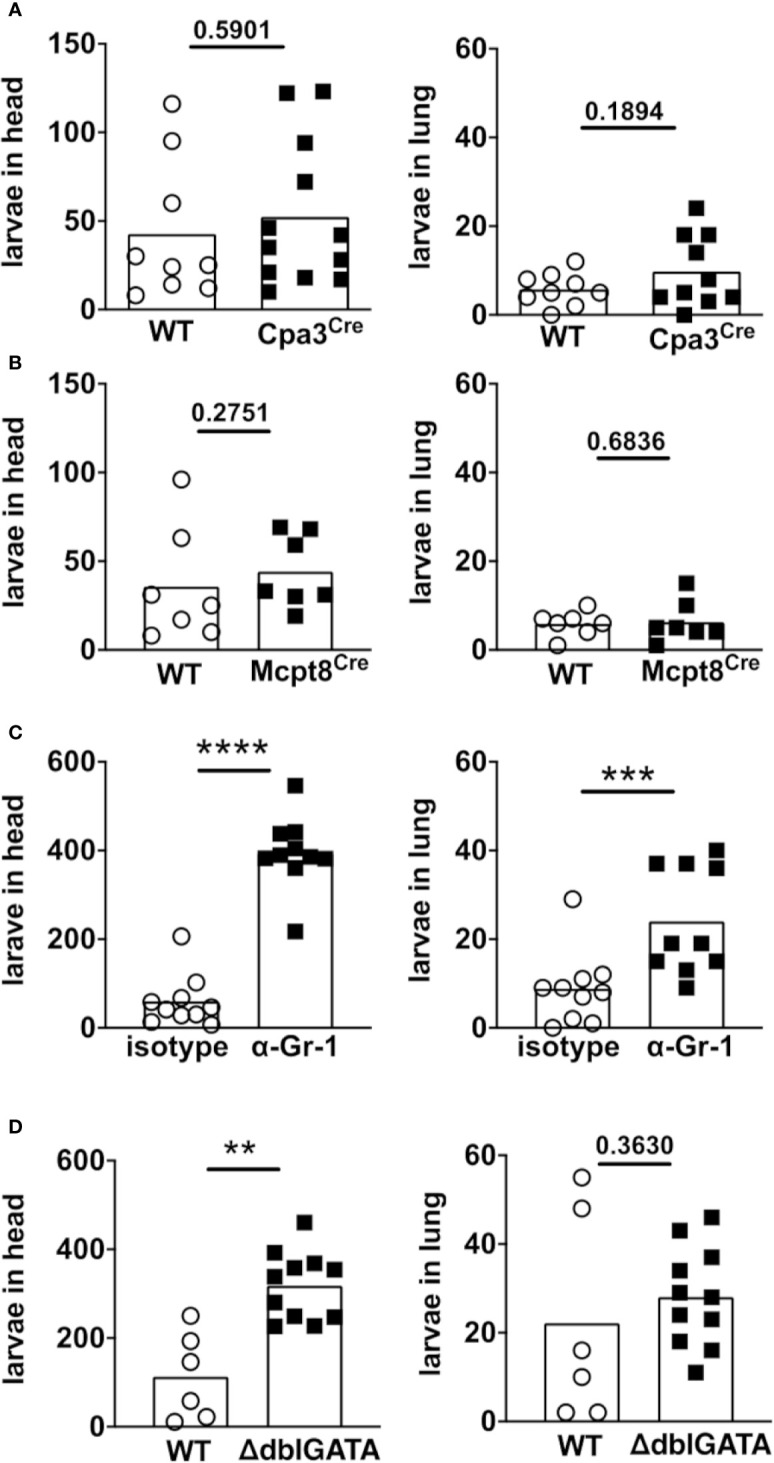
Eosinophils and neutrophils mediate killing of *S. ratti* L3 in the tissue during 1^st^ infection. Mice were infected with 2000 *S. ratti* L3 by s.c. injection into the footpad and sacrificed 2 days later. *S. ratti* L3 that emigrated out of the head and lung tissue were counted. Used were **(A)** mast cell-deficient mice Cpa3^Cre^ mice (black squares) or their Cpa3^WT^ littermates (open circles) or **(B)** basophil-deficient Mcpt8^Cre^ mice (black squares) and Mcpt8^WT^ littermates (open circles) or **(C)** Gr-1^+^ cell-depleted mice (black squares) or isotype-treated (open circles) BALB/c mice or **(D)** eosinophil-deficient ΔdblGATA mice (black squares) and BALB/c WT mice (open circles). Graphs show combined results derived out of 2 independent experiments with 3 - 6 mice per group and experiment, each symbol represents an individual mouse, bars indicate the mean and asterisks significant differences of the mean (students t-test, p < 0.01 **; p < 0.001 ***; p < 0.0001 ****). Gr-1^+^ depletion was controlled day one p.i. and depletion is shown in **Supplementary Figure S1**. Eosinophil and neutrophil accumulation in the skin was seen already after 3 h and 6 h post infection and is shown in **Supplementary Figure S4**.

Since our previous results suggest that migrating *S. ratti* L3 are predominantly trapped and eventually eliminated in skin and muscle tissue surrounding the infection site, we aimed at identifying the responsible effector cell(s). We reasoned that impaired parasite control at the site of infection will lead to increased numbers of viable L3 migrating to lung and head tissue. Therefore, we compared the numbers of L3 in lung and head day 2 p.i. in mice deficient for mast cells, basophils, all Gr-1^+^ cells or eosinophils (**Figure 2**). Absence of mast cells in Cpa3^Cre^ mice (16) did not change numbers of tissue-migrating L3 in head and lung tissue compared to the mast cell-competent Cpa3^WT^ littermates (**Figure 2A**) as we had shown before (20). Likewise, the absence of basophils in Mcpt8^Cre^ mice (17) did not change L3 numbers in the tissue compared to the basophil-competent Mcpt8^WT^ littermates (**Figure 2B**), in line with our earlier findings (21). To deplete all granulocytes including neutrophils, we treated mice with anti Gr-1 mAb (Ly6C/Ly6G) one day before infection with *S. ratti*, resulting in the absence of granulocytes from the peripheral circulation for at least 2 days (**Supplementary Figure S1**). Depletion of Gr-1^+^ cells drastically increased L3 numbers in the head and lung tissue day 2 p.i. (**Figure 2C**). Also, absence of eosinophils in ΔdblGATA mice resulted in a clear and statistically significant increase of L3 numbers in the head while L3 numbers in the lung increased by trend (**Figure 2D**). In summary, these results show that predominantly eosinophils and neutrophils are responsible for the efficient interception of migrating *S. ratti* L3 in the tissue. In line with this reasoning, we detected accumulation of CD45^+^ Ly6G^+^ Ly6C^+^ Siglec F^-^ neutrophils and CD45^+^ Siglec F^+^ eosinophils in the foot and leg-derived skin tissue already at 3 and 6 hours post infection (**Supplementary Figure S4**).

### Neutrophils and Eosinophils Inhibit *S. ratti* L3 Motility *In Vitro*

Since our previous results show that *S. ratti* tissue-migrating L3 are intercepted and eliminated in skin and muscle tissue before arrival in the lung and head tissue (**Figure 1**) by neutrophils and eosinophils (**Figure 2**) we next studied the ability of these cells to immobilize *S. ratti* L3 *in vitro.*

**Figure 3:**
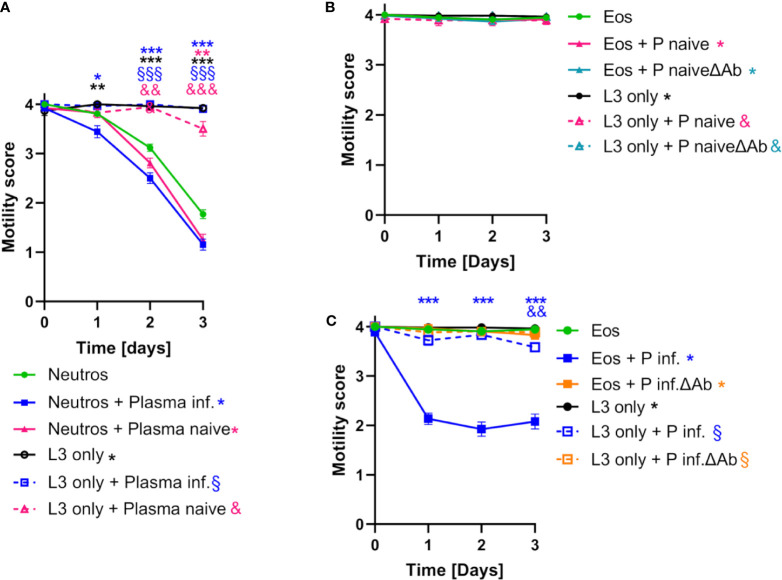
Neutrophils and eosinophils reduce *S. ratti* L3 motility *in vitro.* **(A)** L3 larval motility score of *S. ratti* L3 co-cultured with neutrophils in the absence (•) or presence of plasma from infected (▪) or naive (▲) animals or L3 cultured alone in the absence (L3 only: •) or presence of plasma (infected: □, naive: Δ). Shown are mean with SEM with n=52 per time point and group, of one experiment representative for two independent repeats. Asterisks, § and & show statistical significance calculated by 2-way ANOVA with Bonferroni’s post-hoc test comparing neutrophils with neutrophils + Plasma infected (*), neutrophils + Plasma naive (*), L3 only (*), L3 only + Plasma infected (§) or L3 only + Plasma naive (&). p < 0.05 *, p < 0.01 ** / &&, p < 0.001 *** / &&& / §§§. **(B, C)** L3 motility score co-cultured with eosinophils in the absence (•) or presence of plasma derived from infected or naive animals with (infected: ▪, naive: ▲) or without antibodies (ΔAb) (infected: ▪, naive: ▲) or L3 culture alone in the absence (L3 only: •) or presence of plasma (infected: □;, naive: Δ, infected ΔAb: □, naïve ΔAb: Δ). Shown is mean with SEM with n=52 per time point and group, data are representative for one out of two independent experiments. Asterisk, § and & show statistical significance calculated by 2-way ANOVA with Bonferroni’s post-hoc test comparing eosinophils with eosinophils + Plasma infected (*), eosinophils + Plasma naive (*), eosinophils + Plasma naive ΔAb (*), eosinophils + Plasma infected ΔAb (*), L3 only (*), L3 only + Plasma infected (§) or L3 only + Plasma naive (&), L3 only + Plasma naive Δab (&), L3 only + Plasma infected ΔAb (§). p < 0.01 && p < 0.001 ***. *S. ratti* larval opsonization by immune plasma *in vitro* was determined by fluorescence microscopy and is shown in **Supplementary Figure S5**.

Co-culture of *S. ratti* L3 with bone marrow (BM)-derived neutrophils reduced larval motility by days 2 and 3 without any further stimulus (**Figure 3A**, green squares) compared to *S. ratti* L3 cultured without cells (**Figure 3A**, black circles). Incubation of *S. ratti* L3 with plasma derived from immune mice resulted in strong opsonization by *S. ratti*-specific Ab predominantly of the IgG1 isotype, that are not present in plasma derived from naïve mice (**Supplementary Figure S5**). Addition of immune plasma to the neutrophil/*S. ratti* L3 co-culture led to a modest acceleration of neutrophil activity, as motility inhibition was apparent already at day 1, i.e. one day earlier than in the absence of plasma or in the presence of naïve plasma (**Figure 3A** blue squares to purple and green squares). However, both naïve plasma and immune plasma slightly enhanced the motility inhibiting activity of neutrophils at later time points of the co-culture.

Co-culture of *S. ratti* L3 with BM-derived eosinophils without further stimulus did not inhibit L3 motility (**Figure 3B**, green circles). Addition of immune plasma but not naïve plasma to the co-culture resulted in eosinophil-mediated reduction of L3 motility that was already maximal after 1 day of co-culture (**Figure 3C**, blue squares). This was due to the *S. ratti*-specific Ab present in the immune plasma since depletion of Ab from the immune plasma abrogated the L3 motility inhibition by eosinophils (**Figure 3C**, orange squares). In summary, these data show that BM-derived neutrophils are capable of *S. ratti* L3 motility inhibition *in vitro* without further activation while BM-derived eosinophils inhibit L3 motility selectively in the presence of *S. ratti-*specific Ab.

### *S. ratti* L3 Induce NETosis, as Well as Azurophilic and Tertiary Granule Release by Neutrophils, Which Is Enhanced in the Presence of Plasma

In response to invading helminth larvae, neutrophils are able to release intracellular decondensed DNA that co-localizes with granules containing anti-microbial peptides thus combining the trapping of their “moving target” with killing by direct delivery of molecules with anti-helminth activity (13). Citrullinated histones are frequently detected in neutrophils DNA traps and therefore serve as marker of NETosis (22).

**Figure 4:**
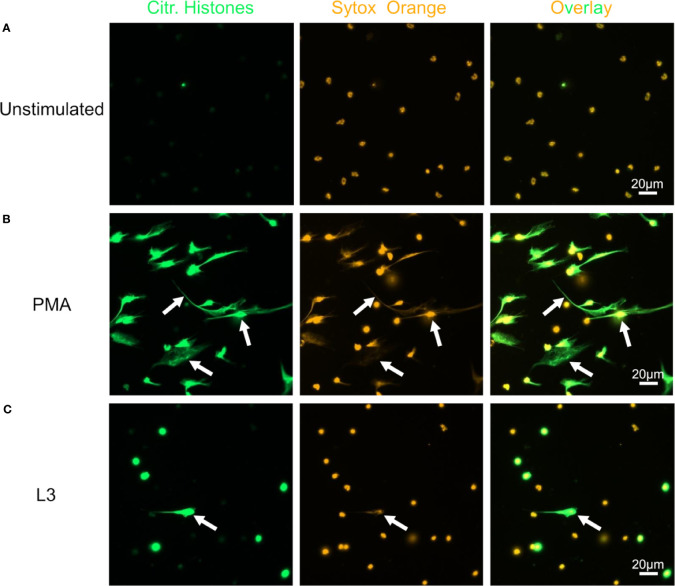
*S. ratti* L3 induce NETosis. Representative fluorescence microscopy pictures of neutrophils left unstimulated **(A)**, stimulated with PMA **(B)** or *S. ratti* L3 larvae **(C)**. Cells were stained for citrullinated histones (green, left pannel) and for DNA with Sytox orange (orange, middle pannel), right pannel shows the overlay. Shown are the results from one out of two independent experiment with n = 2. *S. ratti* larval opsonization by immune plasma *in vitro* was determined by fluorescence microscopy and is shown in **Supplementary Figure S5**.

**Figure 5:**
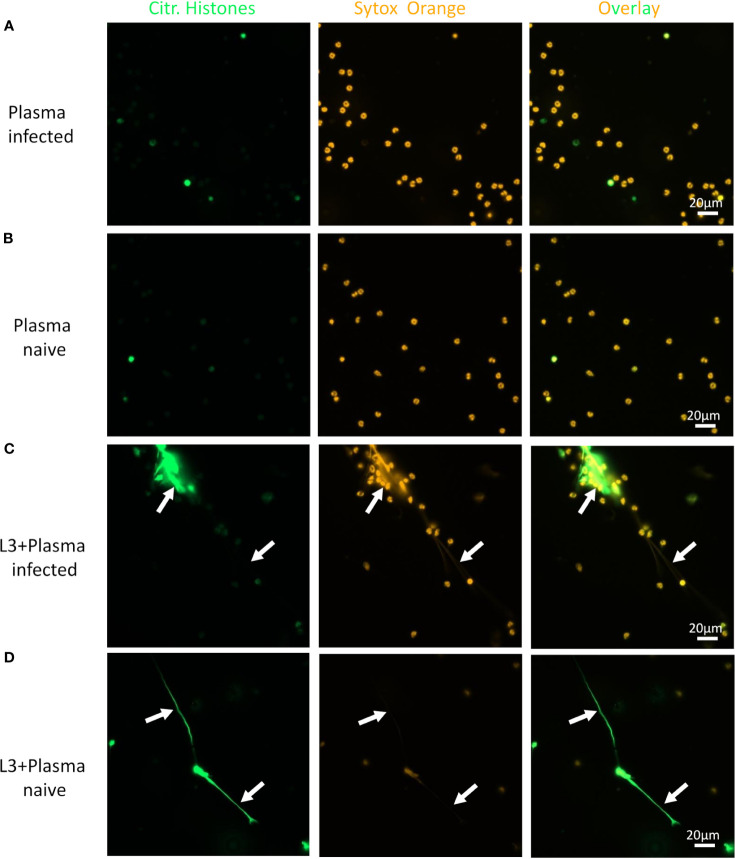
L3-triggered NETosis is enhanced by plasma from infected animals. Representative fluorescence microscopy pictures of neutrophils stimulated with plasma from *S. ratti* infected animals **(A)**, plasma from naive animals **(B)**, *S. ratti* L3 larvae and plasma from *S. ratti* infected animals **(C)** or L3 larvae and plasma from naive animals **(D)**. Cells were stained for citrullinated histones (green, left pannel) and for DNA with Sytox orange (orange, middle pannel), right pannel shows the overlay. Shown are the results from one out of two independent experiment with n = 2.

Neutrophils alone did not release DNA traps (**Figure 4A**), while PMA stimulation readily triggered neutrophil extracellular traps (NET) formation with extracellular, sytox orange-stained DNA traps and massive histone citrullination (**Figure 4B**). Co-culture of neutrophils with L3 induced NETosis by a small number of neutrophils in the absence of other stimuli (**Figure 4C**). Culture of neutrophils alone in the presence of plasma derived from naïve or immune mice did not trigger DNA release (**Figures 5A, B**). Addition of plasma derived from infected mice to co-cultures of L3 and neutrophils strongly triggered NETosis, resulting in formation of large clusters of extracellular DNA, citrullinated histones and neutrophils (**Figure 5C**). Addition of plasma derived from naïve mice to the L3/neutrophil co-cultures triggered modest DNA release (**Figure 5D**).

**Figure 6:**
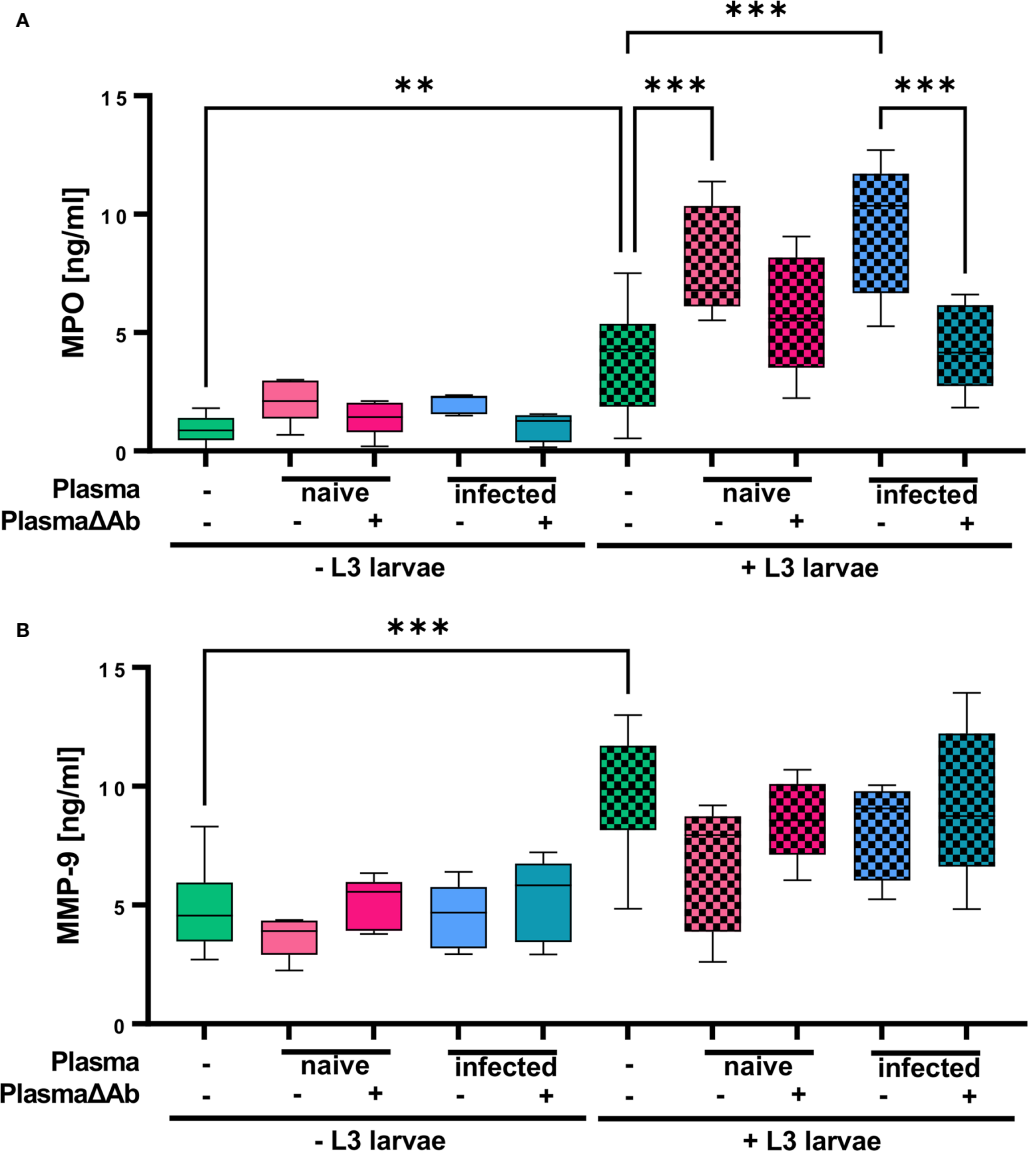
*S. ratti* L3 induce release of MPO and MMP-9-containing granules. **(A)** MPO and **(B)** MMP-9 concentration measured in the supernatant of neutrophils stimulated with plasma from *S. ratti*-infected or naive animals, *S. ratti* L3 larvae alone or in combination with plasma. Plasma has been left untreated or antibody-depleted (ΔAb). Shown are box plots with tukey with n=5. One out of two independent experiment. One-Way ANOVA with Bonferroni *post-hoc* test. Only statistically significant comparisons are depicted, all other comparisons were statistically not significant. p < 0.01 **, p < 0.001 ***.

In addition, MPO derived from primary neutrophil granules as well as MMP-9, stored in tertiary granules, were measured in the supernatant from neutrophil cultures to analyze for specific and unspecific granular release, respectively (**Figure 6**). While plasma alone had no impact on MPO release, L3 triggered a significant increase in MPO release by neutrophils (**Figure 6A**). Addition of both, plasma derived from naïve and from infected mice to the neutrophil/L3 co-culture elevated MPO significantly. Thereby, the increase in MPO release was more pronounced for plasma derived from immune mice containing *S. ratti*-specific Ab than for naïve plasma. Ab-depletion from both, naïve and immune plasma reduced the MPO release by neutrophils compared to plasma that contained Ab to the level of MPO release triggered by L3 alone. This difference was significant for neutrophils stimulated with L3 and plasma from immune mice compared to cells stimulated with L3 and plasma from immune mice without Ab (**Figure 6A**). Similarly, plasma alone had no impact on MMP-9 release, while L3 triggered a significant increase in MMP-9 release by neutrophils (**Figure 6B**). Interestingly, addition of plasma to neutrophil and L3 larval co-culture had no further impact on tertiary granule release (MMP-9) regardless if the plasma was derived from naïve animals or infected animals or was Ab-depleted (**Figure 6B**).

In summary, these results show that neutrophils inhibit L3 motility and produce NETs without further stimuli but especially NETosis and azurophilic granule release, measured by MPO release was strongly enhanced by the presence of *S. ratti* L3-specific Ab.

### Eosinophils Require Ab to Release DNA Traps in Response to *S. ratti* L3

Next to neutrophils, it was also shown that several other immune cells including eosinophils are able to release DNA traps in response to filariae (10, 23, 24). Since eosinophils were responsible for larval entrapment during tissue migration *in vivo* (**Figure 2D**) and larval entrapment *in vitro* (**Figure 3C**), it was investigated if EETosis occurs in response to *S. ratti* L3.

**Figure 7:**
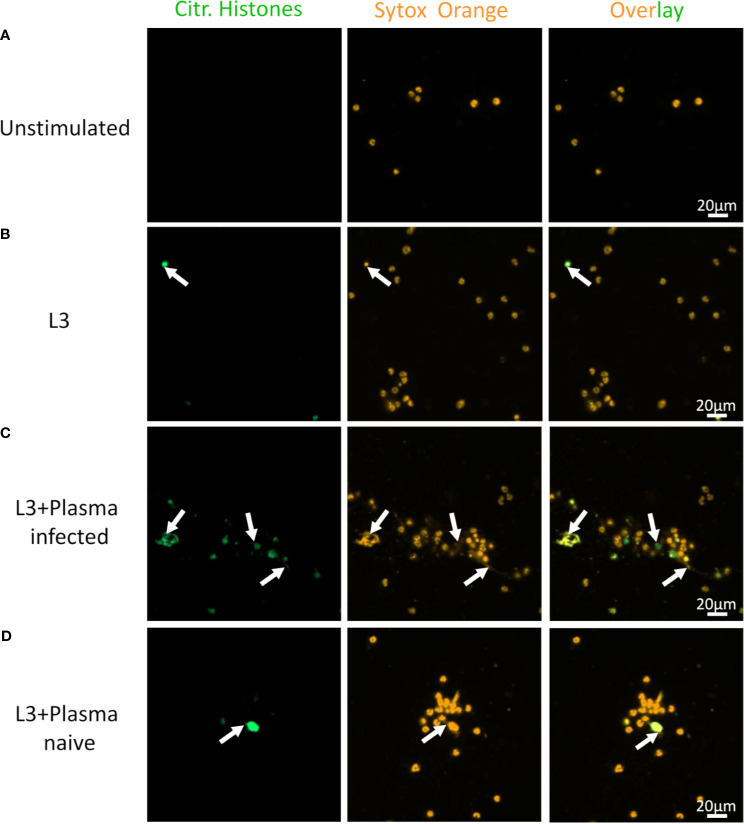
*S. ratti* L3 and Ab induce EETosis. Representative fluorescence microscopy pictures of eosinophils without stimulation **(A)**, stimulated with *S. ratti* L3 alone **(B)** or with plasma from *S. ratti* infected **(C)** or from naive animals **(D)**. Cells were stained for citrullinated histones (green, left pannel) and for DNA with Sytox orange (orange, middle pannel), right pannel shows the overlay. Shown is one out of two independent experiments with n=2.

**Figure 8:**
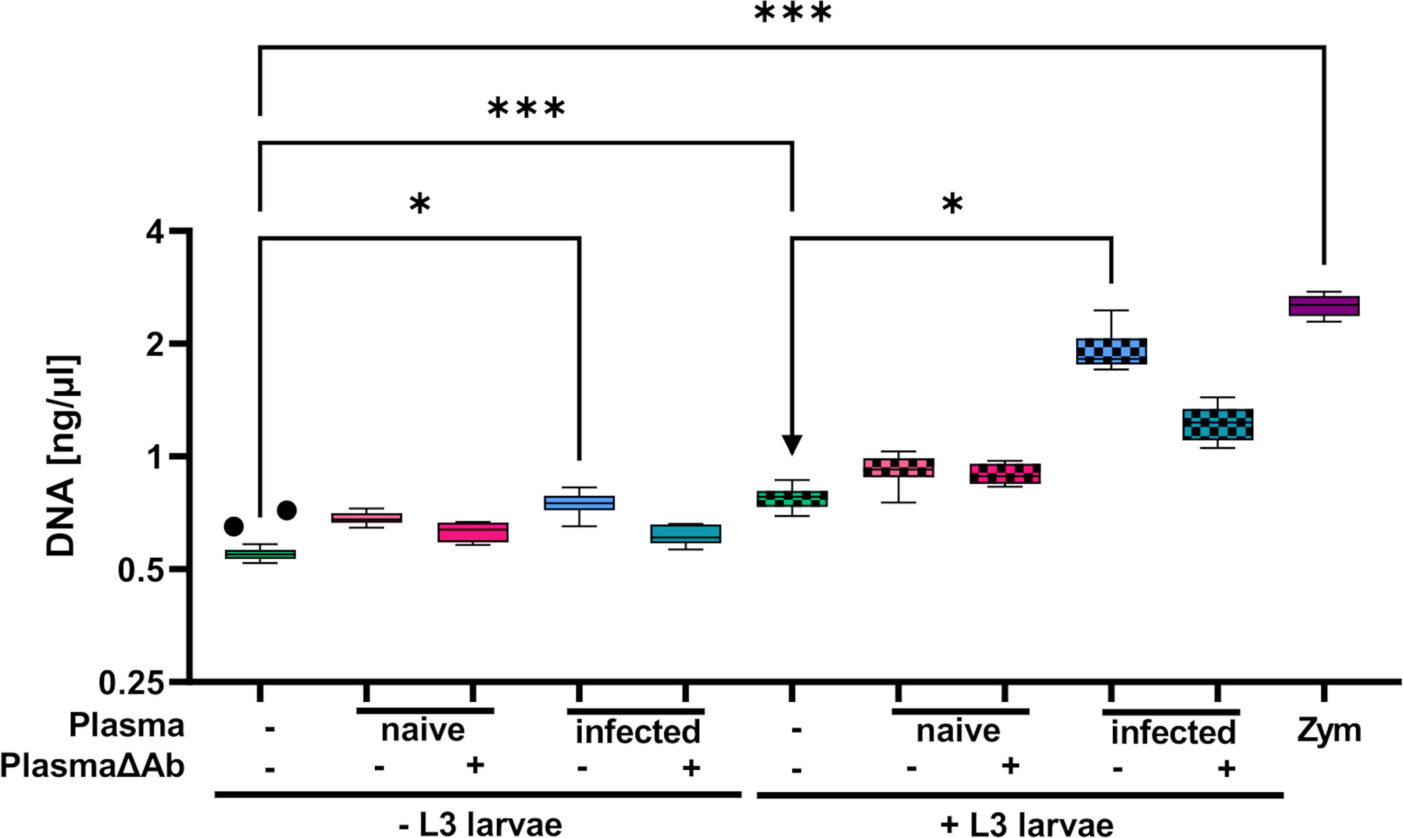
Eosinophils release extracellular DNA (traps) in response to *S. ratti* L3 dependent on immune plasma. DNA quantification in the supernatant of eosinophils in culture with or without *S. ratti* L3 larvae supplemented with or left without plasma from *S. ratti*- infected or naïve animals. Plasma either contained antibodies or was depleted for antibodies (PlasmaΔAb). Shown are box plots with tukey with n=5-20 (n=5: + PlasmaΔAb and + L3 + PlasmaΔAb, n=10: + Plasma and + L3 + Plasma, n=20: unstimulated and + L3 alone). One of two independent experiments. Kruskal-Wallis with Dunn’s *post-hoc* test. Only statistically significant comparisons are depicted, all other comparisons were statistically not significant. p < 0.05 *, p < 0.001 ***.

While eosinophils cultured alone showed no DNA release (**Figure 7A**), addition of L3 triggered histone citrullination in a very low number of eosinophils (**Figure 7B**). Addition of plasma derived from immune (**Figure 7C**) but not from naïve mice (**Figure 7D**) clearly enhanced histone citrullination and trap formation in response to L3. In line with this, DNA quantification of the culture supernatant showed that while plasma alone had no impact on DNA release, L3 led to a modest but statistically significant increased DNA release by eosinophils. Addition of immune plasma but not plasma from naïve animals strongly increased the L3-induced DNA release. Ab depletion from immune plasma resulted in a trend to lower DNA extrusion by eosinophils in response to L3 (**Figure 8**).

In summary, eosinophils, in contrast to neutrophils, displayed rapid L3 motility inhibiting activity selectively in the presence of *S. ratti-*specific Ab and EETosis was enhanced by addition of immune plasma.

## Discussion

### *S. ratti* L3 Are Predominantly Intercepted and Killed in the Skin at the Site of Infection

In the current study, we closely followed the migration of *S. ratti* L3 from the site of entry i.e. the foot, to their final embedding into the small intestine. We provide a tight kinetic of both, the number of viable L3 that were able to emigrate out of the tissue and the amount of larval DNA in each tissue as a rough indicator of total parasite load in a tissue including viable and killed or immobilized larvae.

Tracking viable L3 in mice during a 1^st^ infection showed quick migration from the foot to skin and muscle tissue of the respective leg within 10 minutes, accumulation in the leg for 1 hour and emigration of the majority of L3 after 3 hours out of the leg. We showed that viable L3 arrived subsequently in lung and head tissue with a maximum at day 2 p.i. and further emigrated to the small intestine 3 days after initial infection. Our data comply with early studies reporting a drastic reduction of L3 numbers in the abdominal skin and lung tissue of *S. ratti*-infected immune mice that also suggested improved killing of larvae before the intestinal phase (4). While these studies measured parasite burden using standard emigration assays, in the current study we additionally measured earlier migration kinetics in foot and leg-derived tissues. Comparing *S. ratti*-derived DNA to viable L3, we report that *S. ratti*-derived DNA was detected for 3 days in these tissues, long after viable L3 had left. This finding provides strong evidence that the majority of L3 was killed or retained directly in the foot and leg tissues before migrating further on to the head and lung tissue. The L3 that managed to migrate beyond the foot were not killed in lung and head tissue, as no *S. ratti*-derived DNA was detected in these tissues after emigration of viable L3. Thus, our results strongly suggest that after surviving the first attack in the tissues surrounding the site of entry, the majority of L3 progress to the intestine. This efficient interception of larvae at the site of entry was even more pronounced in immune mice during a 2^nd^ infection where only a negligible number of viable L3 migrated beyond the foot at all.

In line with this model, the skin was shown to be the important site of larval interception in immune mice during a 2^nd^ infection with *N. brasiliensis* (19). Comparable to the *S. ratti* cycle, infective *N. brasiliensis* L3 migrate *via* the skin and later predominantly *via* the lungs to the mouth, are swallowed and subsequently dwell in the intestine (25). The authors elegantly demonstrated that *N. brasiliensis* L3 are efficiently trapped in the skin of immune mice and thus were not detectable in a standard emigration assay. Detection of L3 in the skin of immune mice was possible only after tissue digestion with gastric acid or *via* quantification of larval mRNA, thus highlighting the efficiency of trapping (19). In this study, the interception of *N. brasiliensis* larvae in the skin was only observed in immune mice, during a 2^nd^ infection. In agreement, we observed the almost complete prevention of larval emigration from the foot that prevented lung pathology in immune mice. However, also mice encountering a 1^st^ *S. ratti* infection displayed efficient interception of L3 predominantly in skin and muscle tissue of the foot and the leg.

### Neutrophilic and Eosinophilic Granulocytes Mediate *S. ratti* L3 Killing in the Tissue

Analyzing the innate cell types involved in this interception during a 1^st^ infection *in vivo*, we show that neutrophils and eosinophils accumulated as early as 3 hours post infection in the skin of the infected foot and leg. Neutrophils and eosinophils contributed hereby in a non-redundant manner, as depletion of either Gr1^+^ cells or absence of eosinophils elevated the L3 numbers that migrated beyond the site of entry to the lung and head tissue. Mast cells and basophils, by contrast, were not involved in the control of migrating larvae as their absence did not change L3 numbers in the tissue. This is in line with our previous studies showing that mast cells and basophils predominantly mediate the ejection of *S. ratti* from the intestine but do not contribute to the reduction of parasite burden during tissue migration, neither in naïve nor in immune mice (20, 21).

Our results agree with several studies supporting the role of neutrophils and eosinophils in the control of migrating *Strongyloides* larvae: Neutrophils and eosinophils alongside with macrophages were recruited in response to invading larvae in *S. ratti*-infected mice (26) and a systemic elevation of these cells was observed during *S. venezuelensis* infection (27). Regarding the human pathogenic *S. stercoralis* that cannot patently infect mice, it was shown that *S. stercoralis* extract attracted neutrophils and eosinophils *in vitro* in a CCR3- and CXCR2-dependent manner (28, 29). Analyzing L3 killing in a diffusion chamber implanted into mice it was shown that depletion of eosinophils *via* administration of anti-CCR3 mAb interfered with L3 killing. Addition of eosinophils or neutrophils reciprocally improved L3 killing (30). In the same study also impaired chemotactic recruitment of neutrophils to L3 within diffusion chambers in CXCR2-deficent mice resulted in impaired larval killing in naive and immune mice, thus providing indirect evidence for neutrophil function during primary and secondary infection *in vivo*. Regarding the rodent-specific *S. ratti*, the depletion of Gr1^+^ cells (31) or reduction of eosinophilia by IL-5 depletion (32) were shown to increase *S. ratti* larval numbers in the head at 1.5 days p.i.. In the current study, we reproduced the importance of Gr1^+^ cells in controlling *S. ratti* infection. Gr1 is an epitope that has been shown to be present on Ly6G and Ly6C proteins, which are expressed on several myeloid-derived cells. However, the monoclonal Ab clone RB6.8C5 used in this study has been shown to bind to Ly6G and show no cross-reactivity with Ly6C (33, 34). Thus, this Ab primarily binds neutrophils. It should be noted that a distinct monocyte/macrophage and eosinophil population can be affected by this Ab as well and thus, depletion by the Gr1 Ab may not lead to the exclusive depletion of neutrophils (35).

Regarding eosinophils, we provide the first direct evidence for their non-redundant function in controlling *S. ratti* migration *in vivo* by demonstrating that eosinophil-deficient ΔdblGATA mice displayed increased larval counts in the head. By contrast, killing of *S. stercoralis* larvae within diffusion chambers was unchanged in eosinophil-deficient PHIL mice (36). Since additional depletion of neutrophils elevated larval numbers in the cited study, it was suggested that neutrophils and eosinophils compensated for each other´s absence. This discrepancy may well reflect the different readouts of either measuring survival of *S. stercoralis* larvae that were already “trapped” within an implanted diffusion chamber as opposed to counting larvae that migrated *via* the skin to head and lung tissue. The latter approach clearly results in poor retrieval of the initially injected larvae reflected by the fact that only ~ 300 of the injected 2000 L3 could be counted immediately after infection in the foot. On the other hand, this approach visualizes the interception and killing of migrating larvae, i.e. a “moving target” *in vivo* that would need to be first immobilized as a pre-requisite for the killing later on.

### Killing of *S. ratti* L3 Occurs in the Context of Extracellular DNA Trap Formation and MPO-Containing Granule Release

Explosive release of cellular DNA by neutrophils or NET formation is a central mechanism to combat extracellular pathogens (14, 37, 38) and NET formation in helminth immunity was recently demonstrated with *N. brasiliensis* (14) and *O. volvulus* (12). *S. stercoralis* L3 were shown to trigger NET formation by human and murine bone marrow-derived neutrophils *in vitro* (and *in vivo* in diffusion chambers containing L3 and neutrophils as well as after intraperitoneal injection of L3) (13).

In the current study, we reproduce and extend this finding by showing that neutrophils inhibited the motility of *S. ratti* L3 *in vitro* and displayed limited but significant NET formation without any further stimulus that may reflect direct engagement of pattern recognition receptors by *S. ratti* L3-derived ligands. Addition of plasma from naïve mice slightly and addition of immune plasma that contained L3-opsonizing Ab significantly enhanced NETosis and accelerated L3 motility inhibition. Naïve plasma contains complement that was shown to promote L3 opsonization for neutrophils (39). Thus, neutrophils may initially recognize *S. ratti*-derived pathogen-associated molecular patterns through pattern recognition receptors, while complement components present in naïve plasma as well as *S. ratti-*specific Ab present in the immune plasma enhance neutrophil function.

We have previously shown that eosinophils release extracellular DNA traps in response to *L. sigmodontis* L3 (10). Here, we reveal that eosinophils can undergo EETosis in response to *S. ratti* L3. In contrast to neutrophils, pronounced DNA release by eosinophils was only achieved by L3 in combination with immune plasma. Likewise, L3 motility inhibition by eosinophils was only observed in the presence of immune plasma. Thus, by contrast to neutrophils, larval entrapment by eosinophils occurred in a *S. ratti*-specific Ab-dependent manner and may be correlated to DNA trap formation.

These results highlight the importance of specific Ab and agree with our earlier findings that injection of *S. ratti*-specific immune serum as well as a monoclonal *S. ratti*-specific Ab reduced parasite burden already in the tissue (40). Likewise, the efficient trapping of *N. brasiliensis* in the skin of immune mice was strictly dependent on Ab, although here IgE-activated basophils and subsequent induction of alternatively activated M2 macrophages mediated trapping of larvae in the skin (19). These studies show that Ab-mediated and cell-executed immobilization represents one way of trapping migrating larvae. In addition, the Ab-dependent triggered ETosis by neutrophils and especially by eosinophils that we observed in the current study certainly contributes to larval control in the tissue during 2^nd^ infection. Recently the importance of this defense mechanism was highlighted by the fact that *N. brasiliensis* actively evaded NETosis in immune mice by secretion of a DNase II that degraded DNA traps (14).

While immobilization is the pre-requisite of killing large parasites that cannot be phagocytosed, it is still unclear if trapping within extracellular DNA nets as such would kill larvae. *In vivo* interference with NETosis by DNase treatment or by depletion of Gr1^+^ cells, or by using PAD4-deficient mice or by treating with neutrophil elastase inhibitor, interfered with the killing of *N. brasiliensis* L3 in the tissue as more parasites were recovered in the intestine at 6 days p.i (14). Interference with NETosis also altered migration kinetics leading to a delayed migration of L3 in the absence of neutrophils or upon DNase treatment (14). By contrast, our present study recorded an increased L3 count in head and lung tissues in the absence of neutrophils and eosinophils, thus suggesting that these cells did not modulate migration but clearly prevented the L3 migration at sites upstream of head and lung in the wild-type situation. However, we did not provide a direct causal link between the NETosis and EETosis observed *in vitro* to the central role of neutrophils and eosinophils that we showed *in vivo.* Bonne-Annee and co-workers reported trapping of *S. stercoralis* larvae in NETs that did not always coincide with killing of larvae (13) and hypothesized that immobilization of larvae would allow effector cells to deposit toxic mediators onto the parasites surface more efficiently. Thereby eosinophil-derived MBP and neutrophil-derived MPO were shown to mediate *S. ratti* L3 killing *in vitro* (or in diffusion chambers implanted *in vivo*) in the absence of other cells, while eosinophil peroxidase (EPO) was not essential (36).

In the current study, we observed the release of MPO found in azurophilic granules and MMP-9 from neutrophilic tertiary granules after stimulation. While L3-mediated MPO release was dependent on Ab present in plasma, MMP-9 secretion was not affected by the addition of plasma to the neutrophil and L3 co-culture. Taken together these results show that addition of plasma to neutrophils and L3 results in enhanced DNA trap formation, MPO release and larval motility reduction. Thus, plasma Ab may support MPO-mediated DNA trap formation in response to L3 leading to a synergistically enhanced larval entrapment.

In case of *in vitro* eosinophil and larval co-cultures, immune plasma further raised eosinophil DNA trap formation in an Ab-dependent manner, which could result in the larval entrapment. As those eosinophil DNA traps frequently contain intact granula as well as cytotoxic granula proteins (9), DNA traps not only physically immobilize large pathogens, but also directly deliver those proteins to the pathogen. Future studies should investigate whether the improved elimination of invading L3 upon 2^nd^ infection is due to this enhanced capacity of eosinophils to perform ETosis in the presence of specific Ab.

While the enhanced *in vivo* larval trapping during 2^nd^ infection could be a result of enhanced EETosis, the *in vivo* importance of eosinophils during the 1^st^ infection could not be reflected by the *in vitro* eosinophil culture. Thus, eosinophils prevented L3 migration during primary infection *in vivo*, but *in vitro* larval motility was unaffected by eosinophils alone without immune plasma, i.e. without *S. ratti*-specific Ab that are not present at day 2 of a 1^st^ infection. We speculate that this discrepancy is due to limitations of the *in vitro* assay, which lacks factors present in an *in vivo* setting, e.g. the interaction with additional cell populations or the release of alarmins from somatic cells that could influence eosinophil functions *in vivo*. In addition, eosinophils could support the pathogen recognition and recruitment of other immune cells during a 1^st^ infection *in vivo*. Thus, EETosis-mediated larval trapping may present an additional protective mechanism by eosinophils that further enhances their effector functions during 2^nd^ infection.

Overall, the present study shows that eosinophils and neutrophils play crucial roles in the interception of migrating *S. ratti* larvae at the site of infection. Neutrophils and eosinophils however appear to use different mechanisms in larval entrapment. *In vitro* studies with eosinophils and neutrophils indicate that neutrophils do not require additional plasma-derived factors to entrap L3, although plasma-derived Ab slightly enhanced motility inhibition, DNA trap formation and MPO release. Thus, larval entrapment by neutrophils may occur as an intrinsic effect through NETosis and MPO. Eosinophils on the other hand require *S. ratti-*specific Ab to entrap and immobilize L3s, thus highlighting the potential importance of EETosis especially during a 2^nd^ infection in partially immune mice.

## Concluding Remarks

A better understanding of anti-helminth immunity is the pre-requisite for better and more sustainable treatment of helminth infections that still affect a quarter of the world´s population. Many intestinal helminth parasites such as hookworms display tissue-migrating life-cycle stages and thus face tissue-specific immune defenses before embedding themselves into the intestine. In the current study, we demonstrate the efficiency of larval trapping in skin and muscle tissue at the site of entry. We establish neutrophils and eosinophils as major innate effector cells intercepting larvae *in vivo* and immobilizing larvae *in vitro* and demonstrate NETosis and for the first time EETosis in response to larvae *in vitro* as one possible mechanism of defense.

## Data Availability Statement

The original contributions presented in the study are included in the article/**Supplementary Material**. Further inquiries can be directed to the corresponding authors.

## Ethics Statement

The animal study was reviewed and approved by Federal Health Authorities of the State of Hamburg (permission-numbers N111/16, A029/18 and A20/2020).

## Author Contributions

Conceived and designed the experiments: AE, NR, LH, MH, and MB. Performed the experiments: AE, NR, LH, LL and WH. Analyzed the data: AE, NR, LH, LL and WH. Contributed reagents/materials/analysis tools: MH and MB. Wrote the manuscript: AE, MH, and MB. All authors contributed to the article and approved the submitted version.

## Conflict of Interest

The authors declare that the research was conducted in the absence of any commercial or financial relationships that could be construed as a potential conflict of interest.

## Publisher’s Note

All claims expressed in this article are solely those of the authors and do not necessarily represent those of their affiliated organizations, or those of the publisher, the editors and the reviewers. Any product that may be evaluated in this article, or claim that may be made by its manufacturer, is not guaranteed or endorsed by the publisher.
