# Corrigendum: Eosinophils and Neutrophils Eliminate Migrating *Strongyloides ratti* Larvae at the Site of Infection in the Context of Extracellular DNA Trap Formation

**DOI:** 10.3389/fimmu.2022.878640

**Published:** 2022-03-21

**Authors:** Alexandra Ehrens, Nikolas Rüdiger, Lennart Heepmann, Lara Linnemann, Wiebke Hartmann, Marc P. Hübner, Minka Breloer

**Affiliations:** ^1^ Institute for Medical Microbiology, Immunology and Parasitology, University Hospital Bonn, Bonn, Germany; ^2^ Section of Molecular Biology and Immunology, Bernhard Nocht Institute for Tropical Medicine, Hamburg, Germany; ^3^ German Center for Infection Research (DZIF), Partner site Bonn-Cologne, Bonn, Germany; ^4^ Department of Biology, University of Hamburg, Hamburg, Germany

**Keywords:** nematodes, L3, eosinophils, neutrophils, extracellular DNA traps, ETosis, MPO, *Strongyloides*

In the original article, there was a mistake in **Figure 6** as published. **The units of the axis was indicated in µg/ml and should be corrected to ng/ml**. The corrected **Figure 6** appears below.

**Figure 6:**
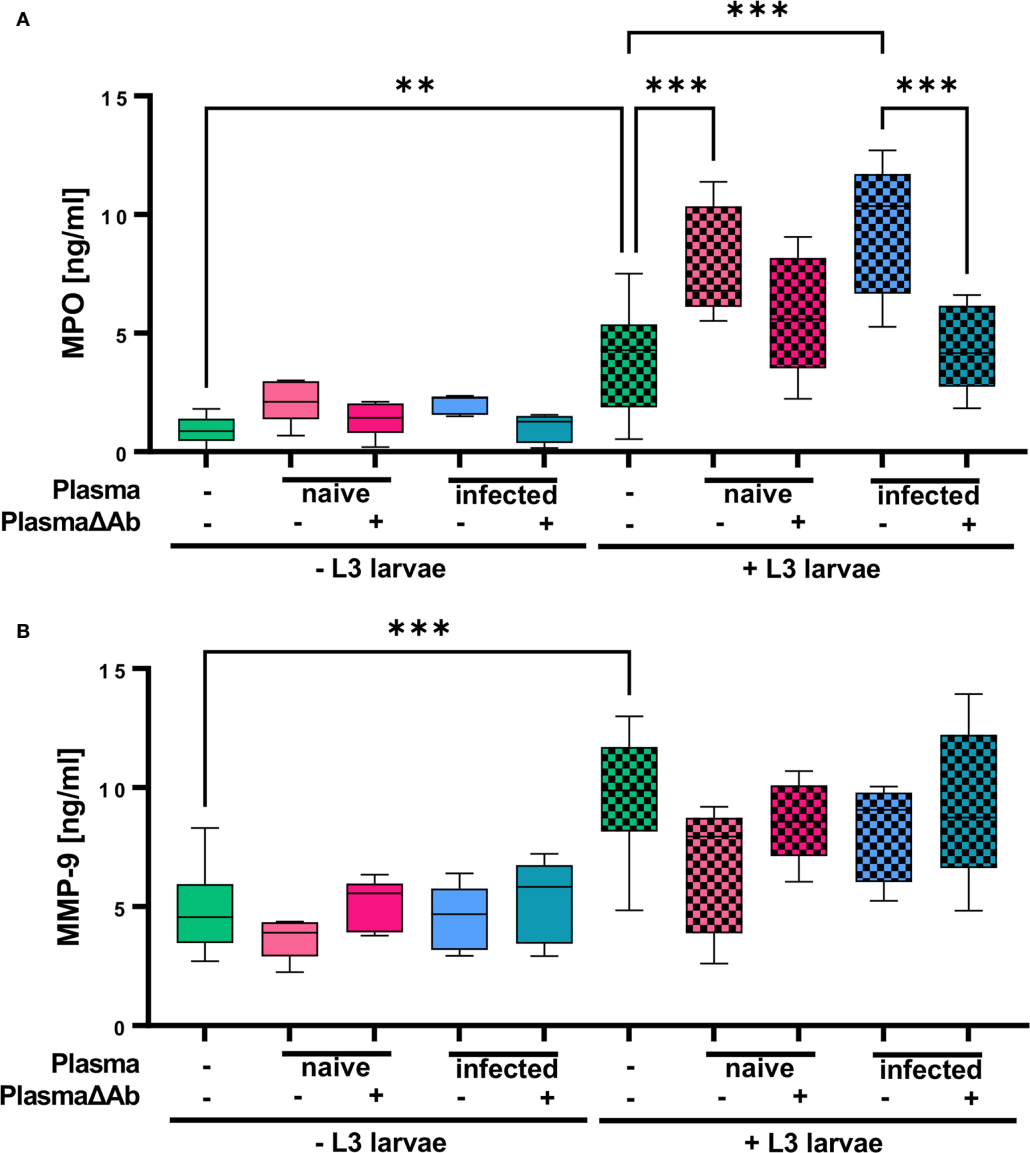
*S. ratti* L3 induce release of MPO and MMP-9-containing granules. **(A)** MPO and **(B)** MMP-9 concentration measured in the supernatant of neutrophils stimulated with plasma from *S. ratti*-infected or naive animals, *S. ratti* L3 larvae alone or in combination with plasma. Plasma has been left untreated or antibody-depleted (ΔAb). Shown are box plots with tukey with n=5. One out of two independent experiment. One-Way ANOVA with Bonferroni post-hoc test. Only statistically significant comparisons are depicted, all other comparisons were statistically not significant. p < 0.01 **, p < 0.001 ***.

The authors apologize for this error and state that this does not change the scientific conclusions of the article in any way. The original article has been updated.

## Publisher’s Note

All claims expressed in this article are solely those of the authors and do not necessarily represent those of their affiliated organizations, or those of the publisher, the editors and the reviewers. Any product that may be evaluated in this article, or claim that may be made by its manufacturer, is not guaranteed or endorsed by the publisher.

