# Supplementary material for: Eosinophils and Neutrophils Eliminate Migrating Strongyloides ratti Larvae at the Site of Infection in the Context of Extracellular DNA Trap Formation
Source: Front Immunol. 2021 Aug 12;12:715766. doi: 10.3389/fimmu.2021.715766 (PMC8406770; doi:10.3389/fimmu.2021.715766)
Supplement: Supplementary file 1 [file DataSheet_1.pdf]

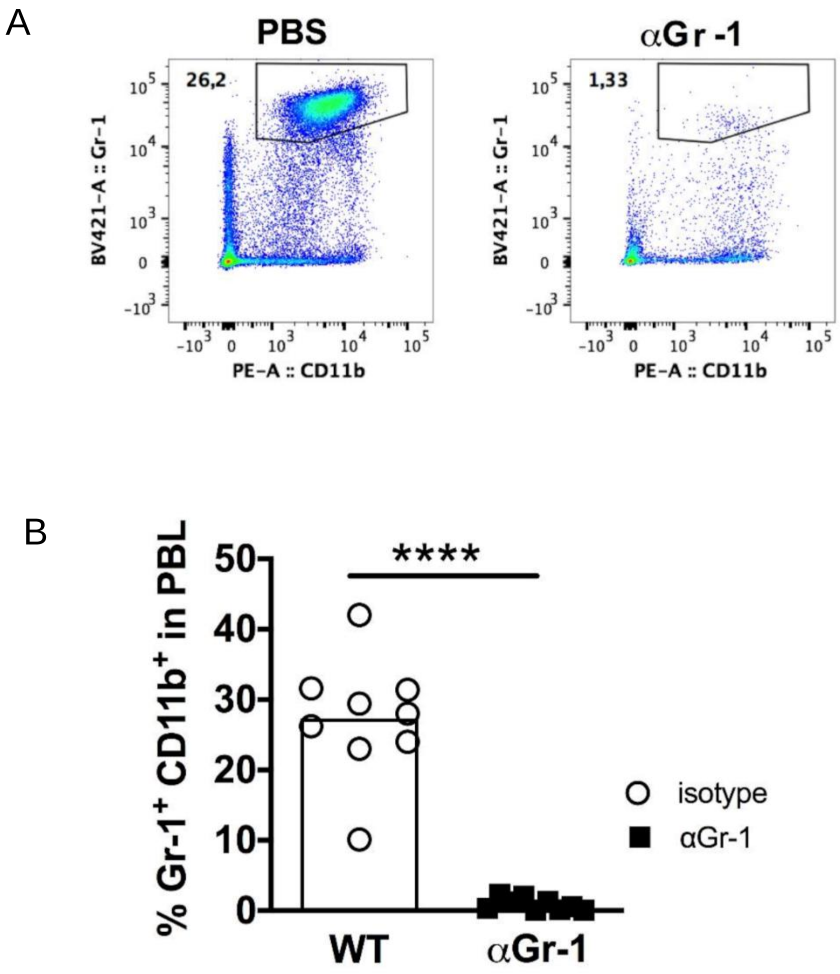

Supplementary Figure S1 (related to Figure 2). Depletion of Gr1$^+$ cells.

BALB/c mice received i.p. 350µg anti-Gr-1 mAb (clone RB6-8C5, black squares) or isotype control (open circles) one day before *S. ratti* infection. Frequency of Gr-1$^+$ CD11b$^+$ cells in the leukocyte gate of PBL were measured by flow cytometry at day 1 p.i.. To this end cells were stained with anti-mouse/human CD11b-PerCP-Cy5.5 (M1/70) and anti-mouse Gr-1-BV421 (RB6-8C5) (BioLegend, Germany), measured with an LSRII Cytometer (BD, Germany) and analyzed by FlowJo software. **(A)** Representative dot plots and **(B)** combined results of 2 independent experiments ($n \geq 4$ per experiment and group) showing frequency of Gr-1$^+$ CD11b$^+$ cells within PBL. Each symbol represents an individual mouse, bars represent the mean and asterisk indicate statistically significant differences of indicated groups. Students t-test. $p<0.0001$****.

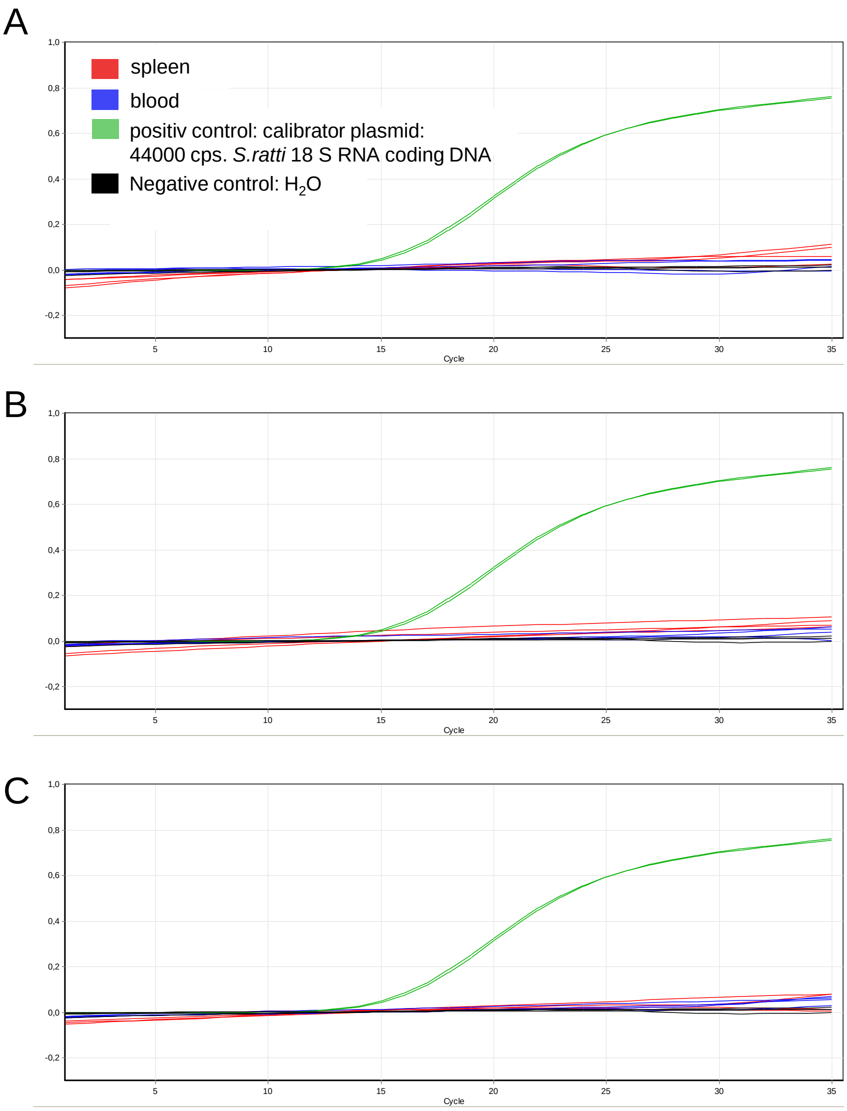

Supplementary Figure S2 (related to Figure 1). No detection of *S. ratti* DNA in blood or spleens of *S. ratti*-infected mice.

Mice were either left naïve **(A)** or were infected with 2000 *S. ratti* L3 s.c. into the footpad and sacrificed after 3 **(B)** or 6 **(C)** hours. Blood was obtained by cardiac puncture and spleens were prepared. Half a spleen and 500-1000 µL blood separated from blood plasma was used for total DNA extraction. *S. ratti*-derived DNA was quantified by qPCR in triplicates as described (6). Shown are normalized fluorescences of qPCR reactions (Y-axis) in relation to cycles (X-axis) of spleen (red), blood (blue), the calibrator plasmid containing a 180 bp fragment of the *S ratti* 18 S RNA gene (green), or water as negative control (black). Each line indicates a single mouse, shown are 4 individual mice per time point and group.

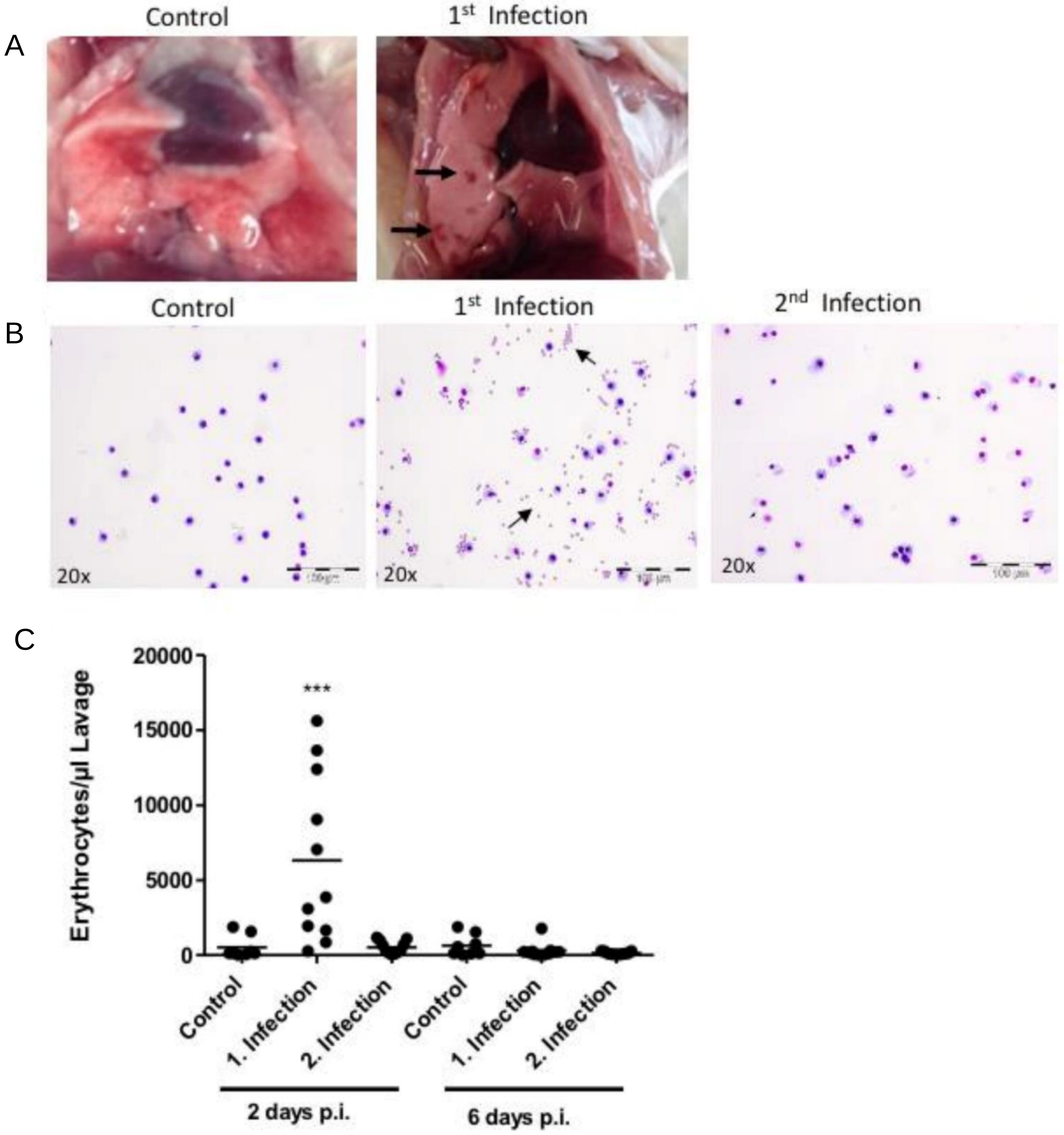

Supplementary Figure S3 (related to Figure 1). Analysis of *S. ratti* lung migration-induced tissue damage.

Naïve and immune BALB/c mice were s.c. infected with 2000 *S. ratti* L3 and sacrificed at indicated time points. **(A)** Representative lung images or **(B)** Representative images of cytospin-prepared BAL, stained with Diff-Quick, derived from uninfected mice (control) or day 2 after a 1st or 2nd *S. ratti* infection. **(C)** Erythrocyte numbers were analyzed in BAL derived from mice during 1st or 2nd *S. ratti* infection at indicated time points. Shown are combined results derived out of 2 experiments with 4-6 mice per group, experiment and time point. Each symbol represents an individual mouse, the line indicates the mean. Asterisks indicate statistically significant difference of the mean compared to control mice 2 days p.i. 1-way ANOVA with Dunnett`s post hoc test. $p<0.001$ ***.

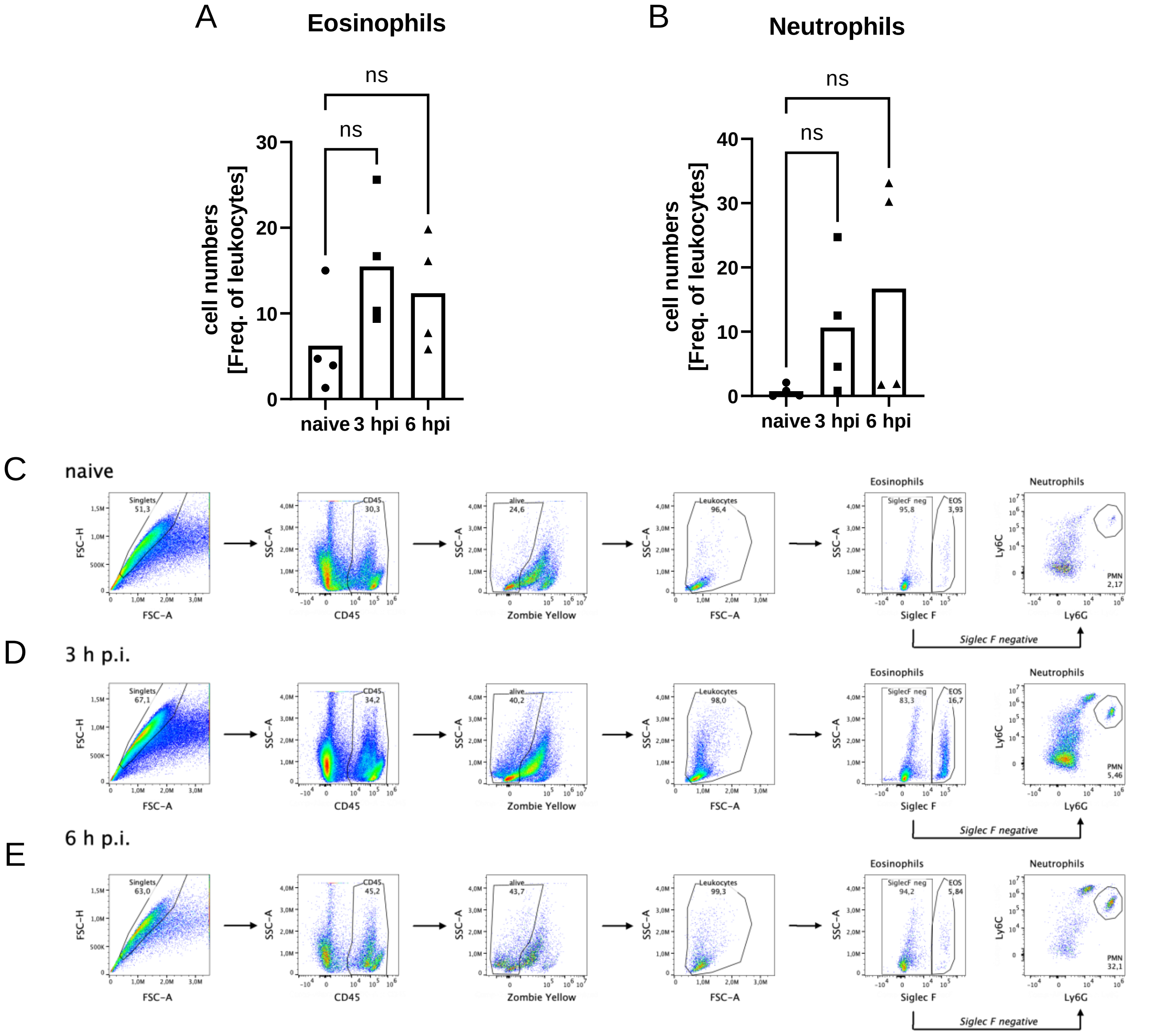

Supplementary Figure S4 (related to Figure 2). Early accumulation of eosinophils and neutrophils in the skin after *subcutaneous S. ratti* primary infection.

Mice were left naïve or were infected with 2000 *S. ratti* L3 into the footpath and analysed at 3 or 6 h *post infection* (p.i.). At the indicated timepoints the mice were sacrificed and the infected lower leg was shaved and amputated. The skin was removed and digested. After exclusion of dead and CD45 negative cells, eosinophils **(A)** were gated as SiglecF$^+$, neutrophils **(B)** as Ly6G$^+$/Ly6C$^+$/SiglecF$^-$ population. Shown is the gating strategy for one representative naïve **(C)** and two infected mice at 3 h p.i. **(D)** and 6 h p.i. **(E)**. Eosinophil and Neutrophil gating. Shown are 2 independent experiments with two mice per group and time point. **(A+B)** Each symbol represents an individual mouse, bars represent the mean. Statistical analysis was done using One-Way ANOVA and Bonferroni post-hoc test after Shapiro-Wilk Normality test. ns= statistically not significant.

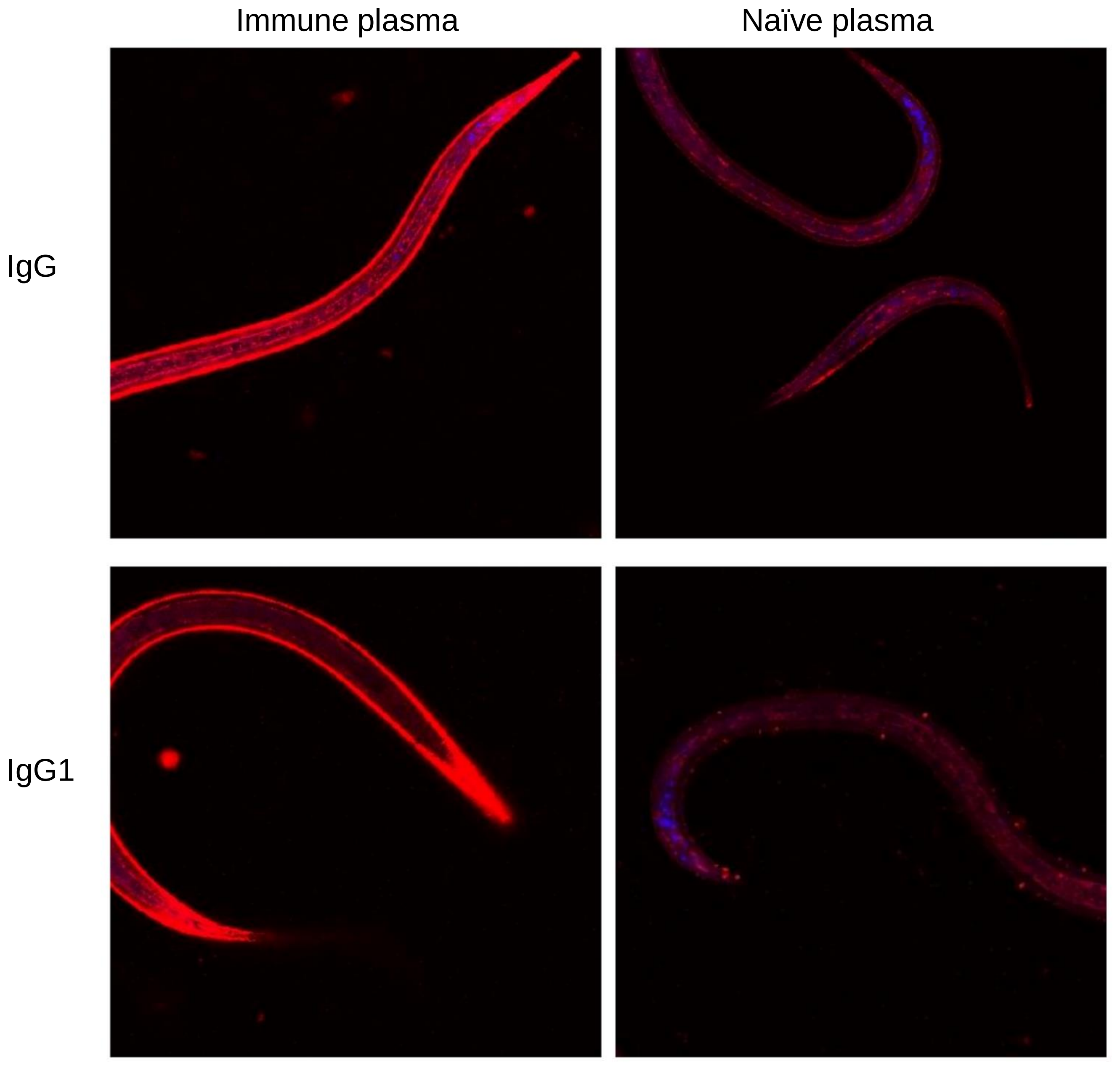

Supplementary Figure S5 (related to Figures 3 and 4). ***S. ratti*** **Larvae is opsonized by immune plasma** ***in vitro.***

To generate *S. ratti* immune plasma, BALB/c mice were s.c. infected with 2000 *S. ratti* L3 and re-infected after 4 weeks with the same dose. Mice were sacrificed day 14 post second infection and blood was collected by cardiac puncture. Plasma of 10 immune mice and 10 naïve mice were pooled. *S. ratti* L3 were incubated with either naïve or immune plasma for 3h at 37°C. Subsequently L3 were fixed with 4% Formalin and stained with anti-mouse IgG A568 or biotinylated anti-mouse IgG1 (both red) and DAPI (blue). Images are representative for 3 cultures of L3 per group.
